# Participant characteristics associated with greater reductions in waist circumference during a four-month, pedometer-based, workplace health program

**DOI:** 10.1186/1471-2458-11-824

**Published:** 2011-10-25

**Authors:** Rosanne LA Freak-Poli, Rory Wolfe, Helen Walls, Kathryn Backholer, Anna Peeters

**Affiliations:** 1 1Department of Epidemiology & Preventive Medicine, Faculty of Medicine, Nursing & Health Sciences, School of Public Health and Preventive Medicine, Monash University, The Alfred Centre, Alfred Hospital, Commercial Road, Melbourne, Victoria 3004, Australia

**Keywords:** waist circumference, workplace, association, prevention, risk-factor, cardiovascular disease, diabetes, health promotion, physical activity, pedometer

## Abstract

**Background:**

Workplace health programs have demonstrated improvements in a number of risk factors for chronic disease. However, there has been little investigation of participant characteristics that may be associated with change in risk factors during such programs. The aim of this paper is to identify participant characteristics associated with improved waist circumference (WC) following participation in a four-month, pedometer-based, physical activity, workplace health program.

**Methods:**

762 adults employed in primarily sedentary occupations and voluntarily enrolled in a four-month workplace program aimed at increasing physical activity were recruited from ten Australian worksites in 2008. Seventy-nine percent returned at the end of the health program. Data included demographic, behavioural, anthropometric and biomedical measurements. WC change (before versus after) was assessed by multivariable linear and logistic regression analyses. Seven groupings of potential associated variables from baseline were sequentially added to build progressively larger regression models.

**Results:**

Greater improvement in WC during the program was associated with having completed tertiary education, consuming two or less standard alcoholic beverages in one occasion in the twelve months prior to baseline, undertaking less baseline weekend sitting time and lower baseline total cholesterol. A greater WC at baseline was strongly associated with a greater improvement in WC. A sub-analysis in participants with a 'high-risk' baseline WC revealed that younger age, enrolling for reasons other than appearance, undertaking less weekend sitting time at baseline, eating two or more pieces of fruit per day at baseline, higher baseline physical functioning and lower baseline body mass index were associated with greater odds of moving to 'low risk' WC at the end of the program.

**Conclusions:**

While employees with 'high-risk' WC at baseline experienced the greatest improvements in WC, the other variables associated with greater WC improvement were generally indicators of better baseline health. These results indicate that employees who started with better health, potentially due to lifestyle or recent behavioural changes, were more likely to respond positively to the program. Future health program initiators should think innovatively to encourage all enrolees along the health spectrum to achieve a successful outcome.

## Background

Workplace health programs have demonstrated improvements in the leading global risk factors for chronic disease [1,2] which has led to their increasing role in chronic disease prevention [1,2]. The majority of research has focused upon evaluating the program outcomes [1,3-7] and program characteristics [2-5,7,8] which have increased the evidence-base for workplace health programs. However, there has been little evaluation of participant characteristics and process indicators that may be related to subsequent change in risk factors during such programs.

Identifying variables associated with a successful outcome in health promotion programs can help to determine the health program's reach and its effectiveness at enrolling a variety of participants, including employees with high health risks. The assessment of variables associated with the program can also be used to identify any inequities in the health program through identification of differences in outcome according to characteristics such as education status. Consequently, variables associated with program success can potentially be used to improve the reach and success of a health program, and this can be achieved by addressing any inequities in the targeting of enrolees or response to the program.

A number of workplace health program evaluations have demonstrated immediate improvements in physical activity, blood pressure and anthropometric measures [9]. As a direct intermediary between physical activity and disease [10-13], waist circumference (WC) can be considered a useful marker of the success of such programs. Only one workplace health program evaluation which considered participant characteristics associated with successful outcomes has been identified [14]. To adequately evaluate variables associated with success in workplace health programs, a comprehensive evaluation needs to be undertaken in a large workplace health program with a range of potential association variables including demographic, behavioural, anthropometric, biomedical and process measures, that can be (where appropriate) assessed in accordance to clinically relevant guidelines. We recently performed an evaluation of a four-month, pedometer-based, workplace health program, with a range of measures at baseline. A pre-post analysis of this program found that WC decreased on average by 1.6 cm [9,15].

The aim of this paper is to identify participant characteristics that are associated with greater improvements in waist circumference (WC) following participation in a four-month, pedometer-based, workplace health program.

## Methods

### Study population

Melbourne workplaces undertaking the 2008 Global Corporate Challenge^® ^(GCC^®^) event were approached to be evaluation sites. Following receipt of the Workplace Consent, employees enrolled in the 2008 GCC^® ^event were approached via email. In early 2008, 762 eligible participants were recruited from ten workplaces, providing a variety of sedentary occupations [9,15]. Seventy-nine percent (n = 604) of participants returned directly after the health program for the four-month data collection [9]. Participants who returned for four-month data collection were less likely to report having diabetes and more likely to be older, participate in the GCC^® ^due to health reasons, be a non-smoker and comply with the health program by undertaking 10,000 daily steps on average [9]. Eighty-eight percent (n = 671) of the total sample at baseline completed the WC measurement. Eighty percent (n = 539) of these participants returned to complete the WC measurement at four-months (89% of the total sample who returned at four-months) [9].

### Description of the program

The GCC^® ^is the provider of a pedometer-based workplace program that is established world-wide and occurs annually. The program involves wearing a visible step-count pedometer with a target of at least 10,000 steps per day for 125 days. Weekly encouragement emails are sent and a website is used for logging daily steps, accessing additional health information, communication amongst participants and comparing team progress. Participation requires an employer or employee financial contribution and is typically competitive.

### Data collection

Data were collected directly prior to the GCC^® ^2008 event (baseline) and immediately after completion of the GCC^® ^2008 event (four-month follow-up). In brief, trained staff visited employees' workplaces for scheduled morning appointments to collect fasting anthropometric and biomedical measurements. Before measurements, participants were asked to remove outer garments, belts and workplace ID tags from around their waists. To record WC, participants were asked to point out their lower rib margin and the top of the hip (iliac crest) and the measurement was taken midway. Waist was recorded using a Figure Finder Tape Measure (Novel Products Inc 2005 code PE024) and a mirror to ensure that the tape was horizontal. An Internet-based self-report questionnaire was completed by participants at their own convenience. The questionnaire incorporated demographic information [16-18], motivation and support for participation, a health history [16] and behavioural measures [16,18,19]. Meeting alcohol guidelines was defined as consuming two or fewer standard drinks on one occasion in the last twelve months [20]. Other national measurement guidelines for risk assessment are summarised in Table 1, including diabetes type 2 and cardiovascular disease predicted risk scores [9,15].

**Table 1:** Guideline recommendation summary table [15]

Guideline recommendation	Not meeting recommended guideline
**BEHAVIOURAL**	
Physical activity^a ^[29-31]	<150mins moderate intensity activity per week
Fruit Intake [29,31,32]	<2 serves per day
Vegetable Intake [29,31,32]	<4 serves per day
Tobacco	≥1 tobacco cigarette(s) per day
**ANTHROPOMETRIC**	
Blood pressure [31,33]	
Systolic	≥ 140 mmHg
Diastolic	≥ 90 mmHg
Body Mass Index (BMI) [31,34-36]	≥25 kg/m^2^
**BIOMEDIAL**	
Fasting Glucose [31,34-36]	≥ 7.0 mmol/L
Cholesterol (total) [37]	≥ 5.5 mmol/L
Triglycerides [38]	≥1.5 mmol/L
**RISK MODELS**	
Diabetes type 2 5-year risk (assessed by The Australian Type 2 Diabetes Risk Assessment Tool - AUSDRISK) [31,39,40]	intermediate 1/100-1/20; high ≥1/20
Cardiovascular disease (CVD) 10-year risk (assessed by the Absolute cardiovascular disease risk assessment tool) [31,41-43]	intermediate 1/100-1/5; high ≥1/5

^a^Physical activity was preferably accrued over at least five sessions per week, with vigorous activity given double weighting

### Step information

Step information was obtained by the GCC^® ^through participation in the program. Participants were asked to enter their step-counts, as indicated on the pedometer, daily into the website diary. Bicycle ride length was also recorded on a daily basis and incorporated into the step-count by the GCC^® ^(6.4 km = 10,000 steps).

Further methodological details of the GCC^® ^Evaluation Study have been described elsewhere [9,15].

### Outcome

WC has been shown to be a better predictor of metabolic risk than body mass index, due to the independent association between an increased WC with health [10-13] and mortality [10,21-25]. Reduction in WC can be achieved through lifestyle changes and is an achievable goal for workplace health program initiatives. Recently it was identified that completion of this four-month, pedometer-based, workplace health program was associated with improvements in WC of 1.6 cm on average [9]. WC change for each participant was calculated by subtracting their baseline measurement from their four-month follow-up measurement.

### Variable selection

All measured variables at baseline, i.e. participant characteristics present prior to commencing the program, were considered potential "predictors" of WC change in the sense that the characteristics pre-dated the program-related changes. The only exception was the process variable, indicating compliance with the program, as measured by step count. Where possible these variables were considered as having a linear continuous-scale relationship with WC change rather than using categorisations that may be arbitrary. Several variables were assessed in accordance to clinically relevant guidelines as summarised in Table 1[9,15].

### Analysis

All analyses were performed using Stata version 11 (Stata Corporation, TX). Robust standard errors, clustered by workplace, were used in all statistical analyses, including the calculation of confidence intervals. A p-value <0.05 was used to determine statistical significance.

Variables related to WC change were assessed by univariable and multivariable linear regression analyses with WC change as the outcome variable. This analysis was repeated in participants with high-risk WC, as defined in Table 1, at baseline that had also completed four-month data collection. Also in participants with high-risk WC at baseline, univariable and multivariable logistic regression analyses were fitted to high/low risk WC at follow-up.

To distinguish associated variables having an indirect influence on WC from participants exerting a more direct influence, two multiple regression approaches were taken. Potential association variables were formed into 10 separate and non-overlapping groups as follows.

1 Age (continuous), sex

2 Socio economic status by SEIFA, tertiary education, occupation and marital status

3 Prior GCC^® ^Event participation and reasons for participation

4 Health behaviours

5 Psychosocial measures

6 Anthropometric measures

7 Biomedical measures

8 Predicted risk scores

9 Workplace characteristics

10 Step-data

In the first approach, the first two groups (baseline demographics) were used for adjustment in separate models containing one of the remaining 8 groups of variables (Multivariable Approach 1 in Tables 2, 3 and 4). In the second approach, these groups of variables were entered sequentially into the regression model, adjusting for the previous group as confounders (Multivariable Approach 2 in Tables 2, 3 and 4). Baseline WC was not included in the models in either approach due to its potential for introducing bias [26]. Consequently, as the diabetes type-2 predicted risk score included WC in the calculation, it was also excluded from the models. Instead, as described above, additional analyses were performed in a subset of participants identified as having high-risk baseline WC.

Glucose and triglyceride variables followed skewed distributions and were log-transformed before inclusion in regression models. Pregnant participants (n = 13) were excluded from analyses. Each analysis used participants with complete data on the relevant variable/s.

### Ethics

The study, project number CF08/0271-2008000125, was approved by Monash University Human Research Ethics through the standing committee on ethics in research-involving humans.

## Results

### Distribution of waist circumference change

**Figure 1:**
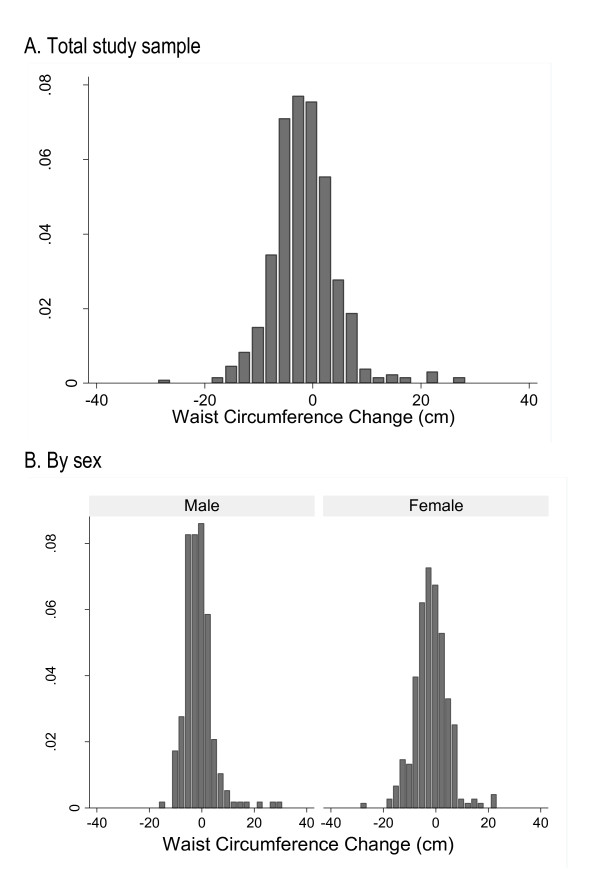
**Distribution of waist circumference change associated with participation in a four-month workplace health program**.

The change in waist circumference (WC) followed an approximately normal distribution with an average reduction of 1.6 cm (±5.9SD), Figure 1a. Males and females were similar in their mean WC reduction (-1.3 ± 5.5SD for males compared to -1.8 ± 6.2SD for females, p = 0.4), Figure 1b.

### Predictors of waist circumference improvement

Between baseline and four-months, variables associated with WC improvement identified through univariable analyses included having completed tertiary education and less weekend or weekday sitting time at baseline, Table 2. Participants who were not meeting guidelines for WC at baseline (as defined in Table 1) responded positively to the program and had a 2.9 cm larger reduction in WC than participants who were meeting guidelines for WC at baseline. For every extra centimetre of WC at baseline, a 0.12 cm loss in WC at four-months was observed. Similarly, participants with high baseline diabetes type-2 risk had a 2.2 cm larger reduction in WC than participants with low baseline diabetes type-2 risk.

**Table 2:** Linear regression analyses assessing potential baseline and step-data predictors of waist circumference change

Group	Predictor Variable	n	Crude WC change	Univariate		**Multivariable Approach 1**^**a**^		**Multivariable Approach 2**^**b**^	
				
				WC change (cm)	P-value	WC change (cm)	P-value	WC change (cm)	P-value
	**DEMOGRAPHICS**								
1	Age (year)	539	-	-0.02	0.5	-0.02	0.5	-0.02	0.5
	Sex								
	Female	305	-1.78	reference		reference		reference	
	Male	234	-1.33	0.45	0.4	0.48	0.4	0.48	0.4
2	Socio Economic Status (by SEIFA %)								
	Most Advantaged	189	-1.46	reference		reference		reference	
	Advantaged	224	-1.50	-0.04	0.9	-0.16	0.7	-0.16	0.7
	Disadvantaged	92	-1.83	-0.37	0.8	-0.49	0.6	-0.49	0.6
	Most Disadvantaged	33	-2.68	-1.21	0.5	-1.07	0.4	-1.07	0.4
	Tertiary Education								
	Not completed	115	-0.04	reference		reference		reference	
	Completed	423	-2.04	-2.00	**0.046**	-2.13	**0.016**	-2.13	**0.016**
	Occupation								
	Professional	221	-1.61	reference		reference		reference	
	Associate professional	97	-2.41	-0.80	0.09	-1.05	0.09	-1.05	0.09
	Manager	96	-1.71	-0.10	0.9	0.07	0.9	0.07	0.9
	Clerical or Service	76	-1.10	0.51	0.7	0.15	0.9	0.15	0.9
	Marital Status								
	Married/de facto	366	-1.38	reference		reference		reference	
	Widowed/separated/divorced	46	-3.21	-1.84	0.07	-1.62	0.2	-1.62	0.2
	Never married	120	-1.85	-0.47	0.7	-0.46	0.6	-0.46	0.6
	**BASELINE MEASURES**								
3	Prior GCC^® ^Participation^c^	532	-1.62	0.08	0.9	0.09	0.8	0.09	0.8
	**Reasons for Participation**^**c**^								
	Health	531	-1.94	-0.93	0.09	-0.71	0.3	-0.71	0.3
	To look my best	531	-1.82	-0.45	0.1	-0.27	0.3	-0.27	0.3
	Fitness	531	-1.75	-0.36	0.2	-0.09	0.7	-0.09	0.7
	Colleagues	531	-1.72	-0.20	0.6	-0.24	0.7	-0.24	0.7
	Friends or family	531	-2.14	-0.52	0.6	-0.92	0.4	-0.92	0.4
	Behavioural Measures								
4	Fruit Intake								
	Not meeting guidelines	364	-1.21	reference					
	Meeting guidelines	168	-2.58	-1.37	0.06	-1.04	0.09	-1.01	0.1
	Vegetable Intake								
	Not meeting guidelines	455	-1.49	reference					
	Meeting guidelines	77	-2.56	-1.08	0.06	-0.38	0.6	-0.51	0.5
	Takeaway Dinner								
	Once or less per month	246	-1.68	reference					
	About once a week	214	-1.63	0.05	0.9	-0.70	0.2	-0.62	0.3
	More than once a week	72	-1.56	0.12	0.9	-0.67	0.6	-0.61	0.6
	Alcohol								
	Not meeting guidelines	307	-1.42	reference					
	Meeting guidelines	225	-1.95	-0.54	0.2	-0.58	**0.035**	-0.65	**0.016**
	Tobacco								
	Smoker	50	-1.00	reference					
	Non-smoker	482	-1.71	-0.71	0.4	0.13	0.9	0.32	0.7
	Physical Activity								
	Not meeting guidelines	322	-1.49	reference					
	Meeting guidelines	209	-1.87	-0.38	0.5	0.00	1.0	-0.10	0.9
	Sitting Time (per hour per day)								
	Weekday	530	-	0.19	**0.015**	0.09	**0.048**	0.10	0.06
	Weekend	529	-	0.12	**0.026**	0.28	**0.018**	0.28	**0.025**
	**Psychosocial Measures**								
5	Physical Functioning (SF12)	523	-	-0.06	0.09	-0.05	0.08	-0.05	0.2
	Mental Functioning (SF12)	523	-	0.00	0.9	-0.02	0.6	-0.01	0.5
	**Anthropometric Measures**^**d**^								
6	Blood Pressure (mmHg)								
	Systolic	517	-	-0.01	0.5	0.00	1.0	0.00	0.9
	Diastolic	517	-	0.03	0.2	-0.03	0.6	-0.03	0.5
	Not meeting guidelines	90	-1.72	reference					
	Meeting guidelines	427	-1.51	0.20	0.6				
	Heart rate (Mean, SD)	517	-	-0.03	0.3	-0.02	0.5	-0.04	0.2
	Weight (Mean, SD)^e^	537	-	-0.02	0.4				
	Body Mass Index								
	Body Mass Index	222	-	-0.07	0.08	-0.08	0.08	-0.08	0.08
	Not meeting guidelines	315	-1.68	reference					
	Meeting guidelines	222	-1.51	0.17	0.8				
	Waist circumference^f^								
	Waist circumference	539	-	-0.12	**0.001**				
	Not meeting guidelines	288	-2.86	reference					
	Meeting guidelines	251	-0.12	2.74	**0.001**				
	**Biomedical Measures (fasting)**^**d**^								
7	Total Cholesterol								
	Total Cholesterol	535	-	0.20	0.4	0.49	**0.034**	0.38	**0.029**
	Not meeting guidelines	150	-1.66	reference					
	Meeting guidelines	385	-1.63	0.03	1.0				
	Glucose								
	Glucose	535	-	0.12	0.3	0.06	0.2	0.02	0.09
	Not meeting guidelines	20	0.05	reference					
	Meeting guidelines	535	1.49	4.23	0.3				
	Triglycerides								
	Triglycerides	535	-	0.83	0.7	0.49	0.1	0.99	1.0
	Not meeting guidelines	109	0.10	reference					
	Meeting guidelines	426	2.43	2.33	0.1				
	**Predicted risk scores**^**d**^								
8	Cardiovascular disease risk (next 10 years)								
	CVD risk (continuous)	504	-	-0.03	0.6	-0.08	0.2	-0.11	0.4
	Low-risk	452	-1.61	reference					
	Intermediate-risk	45	-1.76	-0.14	0.9				
	High-risk	7	-2.91	-1.30	0.056				
	Diabetes risk (next 5 years)^f^								
	Diabetes risk (continuous)	529	-	-0.09	0.09				
	Low-risk	202	-1.26	reference					
	Intermediate-risk	276	-1.55	-0.28	0.6				
	High-risk	51	-3.45	-2.19	**0.004**				
	**WORKPLACE CHARACTERISTICS**^**g**^								
9	Public ownership (vs. private)	539	-2.00	-0.67	0.4	-0.57	0.2	-0.37	0.5
	Outer city location (vs. inner city)	539	-2.22	-0.37	0.7	0.16	0.8	0.06	1.0
	**PROCESS MEASURES**								
	**Step data**^**d**^								
10	Step average per day (per 10,000 steps)	538	-	-0.20	0.7	-0.25	0.8	0.12	0.9
	<10,000 steps average (per day)	179	-1.48	reference					
	Meeting ≥10,000 steps average (per day)	359	-1.65	-0.17	0.7				

^a ^Associated risk-factor subgroups adjusted only for baseline demographic confounders

^b ^Associated risk-factor subgroups adjusted for all potential predictors within the analysis step and above

^c ^The reference group for this binary variable is 'no'. The reference group data is not shown.

^d ^Continuous variables chosen over categorical variables

^e ^Excluded due to the inclusion of body mass index (BMI)

^f ^Excluded from model as baseline WC was not considered as a predictor.

^g ^The reference group for this binary variable is shown in brackets. The reference group data is not shown.

Multivariable approaches 1 and 2 produced similar results, Table 2. Between baseline and four-months, participants who had completed tertiary education at baseline had a 2.1 cm larger reduction in WC than participants who had not completed tertiary education at baseline. On average, participants who reported at baseline that they did not consume more than two alcoholic standard drinks in one occasion in the past twelve months reduced their WC by 0.7 cm more than participants who did not meet alcohol recommended guidelines. For every hour less per day of baseline weekend sitting time, a 0.3 cm greater loss in WC at four-months was observed. For every millimole per litre less of total cholesterol at baseline, a 0.4 cm greater loss in WC at four-months was observed.

### High-risk versus low-risk baseline waist circumference

To investigate whether variables associated with WC change alter for participants most at risk, further analyses were undertaken in this sub-group. Fifty-three percent (n = 288) of participants were categorised as having a high-risk WC at baseline. Participants who had a high-risk WC at baseline were more likely to be older; be female; have a managerial, clerical or service occupation; work for a publically owned company; participate in the program due to health, fitness or appearance reasons; and have poorer health indicators when compared to baseline low-risk WC participants who returned at four-months, Table 3.

**Table 3:** Linear regression assessing baseline variables associated with WC change in participants not meeting WC guidelines

Group	Predictor Variable	n	Crude WC change	Univariate		**Multivariable Approach 1**^**a**^		**Multivariable Approach 2**^**b**^	
				
				WC change (cm)	P-value	WC change (cm)	P-value	WC change (cm)	P-value
	**DEMOGRAPHICS**								
1	Age (year)	288	-	0.03	0.4	0.03	0.4	0.03	0.4
	Sex								
	Female	185	-2.94	reference		reference		reference	
	Male	103	-2.73	0.21	0.6	0.16	0.7	0.16	0.7
2	Socio Economic Status (by SEIFA %)								
	Most Advantaged	95	-2.84	reference		reference		reference	
	Advantaged	122	-2.61	0.23	0.8	0.32	0.6	0.32	0.6
	Disadvantaged	52	-2.82	0.02	1.0	0.31	0.9	0.31	0.9
	Most Disadvantaged	18	-5.78	-2.94	0.1	-2.11	0.3	-2.11	0.3
	Tertiary Education								
	Not completed	69	-1.08	reference		reference		reference	
	Completed	219	-3.43	-2.35	**0.018**	-2.35	**0.017**	-2.35	**0.017**
	Occupation								
	Professional	105	-2.34	reference		reference		reference	
	Associate professional	53	-4.13	-1.79	0.06	-1.89	0.1	-1.89	0.1
	Manager	55	-3.52	-1.18	0.08	-1.00	0.2	-1.00	0.2
	Clerical or Service	48	-2.84	-0.50	0.7	-1.06	0.4	-1.06	0.4
	Marital Status								
	Married/de facto	200	-2.45	reference		reference		reference	
	Widowed/separated/divorced	26	-4.82	-2.37	0.2	-2.42	0.2	-2.42	0.2
	Never married	60	-3.48	-1.02	0.3	-0.36	0.6	-0.36	0.6
	**BASELINE MEASURES**								
3	Prior GCC^® ^Participation^c^	286	-2.86	0.08	0.8	-0.04	0.9	-0.04	0.9
	**Reasons for Participation**^**c**^								
	Health	286	-2.82	0.27	0.8	0.14	0.9	0.14	0.9
	To look my best	286	-2.80	0.32	0.5	0.32	0.6	0.32	0.6
	Fitness	286	-2.75	0.49	0.4	0.07	0.9	0.07	0.9
	Colleagues	286	-3.24	-0.81	0.053	-0.72	0.3	-0.72	0.3
	Friends or family	286	-4.01	-1.15	0.2	-1.10	0.3	-1.10	0.3
	**Behavioural Measures**								
4	Fruit Intake								
	Not meeting guidelines	188	-2.42	reference					
	Meeting guidelines	98	-3.77	-1.34	**0.027**	-0.96	0.3	-0.96	0.3
	Vegetable Intake								
	Not meeting guidelines	244	-2.77	reference					
	Meeting guidelines	42	-3.52	-0.75	0.4	0.40	0.8	0.32	0.8
	Takeaway Dinner								
	Once or less per month	132	-0.55	reference					
	About once a week	117	-1.34	-0.79	0.2	-0.63	0.2	-0.52	0.2
	More than once a week	37	-3.11	-2.55	0.7	-0.85	0.7	-0.73	0.7
	Alcohol								
	Not meeting guidelines	159	-2.95	reference					
	Meeting guidelines	127	-2.80	0.15	0.8	-0.28	0.6	-0.28	0.6
	Tobacco								
	Smoker	32	-1.30	reference					
	Non-smoker	254	-3.03	-1.73	0.2	0.38	0.7	0.49	0.6
	Physical Activity								
	Not meeting guidelines	178	-2.45	reference					
	Meeting guidelines	108	-3.60	-1.15	0.2	-0.92	0.3	-0.94	0.3
	Sitting Time (per hour per day)								
	Weekday	286	-	0.15	0.2	0.11	0.4	0.12	0.4
	Weekend	282	-	0.26	0.2	0.23	0.3	0.23	0.3
	**Psychosocial Measures**								
5	Physical Functioning (SF12)	282	-	-0.11	**0.049**	-0.09	**0.02**	-0.07	0.2
	Mental Functioning (SF12)	282	-	0.00	1.0	-0.03	0.3	-0.02	0.5
	**Anthropometric Measures**^**d**^								
6	Blood Pressure (mmHg)								
	Systolic	273	-	0.02	0.4	0.00	1.0	0.00	0.9
	Diastolic	273	-	0.02	0.6	-0.01	0.8	-0.02	0.6
	Not meeting guidelines	56	-2.21	reference					
	Meeting guidelines	217	-3.03	-0.82	0.3				
	Heart rate (Mean, SD)	288	-	-0.02	0.6	-0.02	0.7	-0.02	0.7
	Weight (Mean, SD)^e^	288	-	0.02	0.5				
	Body Mass Index								
	Body Mass Index	288	-	0.13	0.2	0.12	0.3	0.08	0.5
	Not meeting guidelines	238	-2.49	reference					
	Meeting guidelines	50	-4.63	-2.14	0.1				
	Waist circumference^f^								
	Waist circumference	288	-	-0.05	0.2				
	Not meeting guidelines	288	n/a						
	Meeting guidelines	0							
	**Biomedical Measures (fasting)**^**d**^								
7	Total Cholesterol								
	Total Cholesterol	285	-	0.47	0.2	0.79	0.051	0.86	**0.014**
	Not meeting guidelines	89	-2.71	reference					
	Meeting guidelines	196	-2.94	-0.23	0.7				
	Glucose								
	Glucose	285	-	2.29	0.8	0.22	0.6	0.03	0.3
	Not meeting guidelines	18	0.06	reference					
	Meeting guidelines	267	1.07	1.02	1.0				
	Triglycerides								
	Triglycerides	285	-	1.31	0.6	0.47	0.2	0.45	0.2
	Not meeting guidelines	73	0.03	reference					
	Meeting guidelines	212	1.96	1.93	0.4				
	**Predicted risk scores**^**d**^								
8	Cardiovascular disease risk (next 10 years)								
	CVD risk (continuous)	267	-	0.09	0.2	0.03	0.7	0.02	0.9
	Low-risk	227	-2.89	reference					
	Intermediate-risk	33	-2.79	0.09	0.9				
	High-risk	7	-2.91	-0.03	1.0				
	Diabetes risk (next 5 years)^f^								
	Diabetes risk (continuous)	285	-	0.06	0.3				
	Low-risk	67	-3.79	reference					
	Intermediate-risk	173	-2.34	1.45	**0.033**				
	High-risk	45	-3.55	0.24	0.6				
	**WORKPLACE CHARACTERISTICS**^**g**^								
9	Public ownership (vs. private)	288	-2.66	0.36	0.6	0.09	0.9	0.06	0.9
	Outer city location (vs. inner city)	288	-3.08	-0.68	0.4	-0.96	0.3	-1.34	0.2
	**PROCESS MEASURES**								
	**Step data**^**d**^								
10	Step average per day (per 10,000 steps)	288	-	-1.04	0.08	-1.40	0.07	-0.55	0.6
	<10,000 steps average (per day)	190	-2.13	reference					
	Meeting ≥10,000 steps average (per day)	190	-3.24	-1.11	**0.049**				

^a ^Associated risk-factor subgroups adjusted only for baseline demographic confounders

^b ^Associated risk-factor subgroups adjusted for all potential predictors within the analysis Group and above

^c ^The reference group for this binary variable is 'no'. The reference group data is not shown.

^d ^Continuous variables chosen over categorical variables

^e ^Excluded due to the inclusion of body mass index (BMI)

^f ^Excluded from model as baseline WC was not considered as a predictor.

^g ^The reference group for this binary variable is shown in brackets. The reference group data is not shown.

Note: For participants not meeting WC guidelines at baseline

### Predictors of continuous waist circumference improvement in participants with high-risk waist circumference at baseline

Within the baseline high-risk WC group, variables associated with WC improvement identified through univariable analyses included having completed tertiary education, eating two or more serves of fruit per day, having greater physical functioning at baseline and meeting the goal of at least 10,000 steps per day on average during the health program, Table 4. Within the high WC risk baseline group, a weak association between baseline WC and improved WC was observed, however it was not statistically significant nor was it of the same magnitude of the observed association in all participants. An additional analysis within the high-risk WC group comparing the BMI in the obese group to the BMI in the normal group in regards to WC change indicated weak evidence of a relationship (2.8 cm greater loss in the normal weight group, p = 0.06). Participants with moderate baseline diabetes type-2 risk had a 1.4 cm larger reduction in WC when compared to participants with low baseline diabetes type-2 risk, however no relationship with high diabetes type-2 risk was identified.

**Table 4:** Assessment of variables associated with improving WC to meet recommended guidelines at follow-up

Group	Predictor Variable	n	% meeting WC guidelines at four-months	Univariate		**Multivariable Approach 1**^**a**^		**Multivariable Approach 2**^**b**^	
				
				OR	P-value	OR	P-value	OR	P-value
	**DEMOGRAPHICS**								
1	Age (year)	288	-	0.97	0.08	0.97	**0.039**	0.97	**0.039**
	Sex								
	Female	185	21.08	reference		reference		reference	
	Male	103	26.21	1.33	0.4	1.43	0.3	1.43	0.3
2	Socio Economic Status (by SEIFA %)								
	Most Advantaged	95	21.05	reference		reference		reference	
	Advantaged	122	22.13	1.07	0.9	1.03	0.9	1.03	0.9
	Disadvantaged	52	26.92	1.38	0.6	1.39	0.5	1.39	0.5
	Most Disadvantaged	18	27.78	1.44	0.4	1.63	0.2	1.63	0.2
	Tertiary Education								
	Not completed	69	20.29	reference		reference		reference	
	Completed	219	23.74	1.22	0.3	1.07	0.8	1.07	0.8
	Occupation								
	Professional	105	22.86	reference		reference		reference	
	Associate professional	53	22.64	0.99	1.0	0.96	0.9	0.96	0.9
	Manager	55	23.64	1.04	0.9	1.04	0.9	1.04	0.9
	Clerical or Service	48	27.08	1.25	0.4	1.51	0.2	1.51	0.2
	Marital Status								
	Married/de facto	200	23	reference		reference		reference	
	Widowed/separated/divorced	26	23.08	1.00	1.0	1.34	0.7	1.34	0.7
	Never married	60	23.33	1.02	1.0	0.71	0.4	0.71	0.4
	**BASELINE MEASURES**								
3	Prior GCC^® ^Participation^c^	286	22.54	0.96	0.9	0.88	0.6	1.13	0.6
	**Reasons for Participation**^**c**^								
	Health	286	21.92	0.76	0.4	1.22	0.6	1.22	0.6
	To look my best	286	19.23	0.48	**<0.001**	0.38	**0.004**	0.38	**0.004**
	Fitness	286	20.57	0.61	0.2	0.93	0.9	0.93	0.9
	Colleagues	286	23.9	1.11	0.7	0.89	0.7	0.89	0.7
	**Friends or family**	286	14.29	0.55	0.6	0.69	0.8	0.69	0.8
	**Behavioural Measures**								
4	Fruit Intake								
	Not meeting guidelines	188	19.15	reference					
	Meeting guidelines	98	30.61	1.86	**0.028**	2.42	**<0.001**	3.05	**0.001**
	Vegetable Intake								
	Not meeting guidelines	244	23.36	reference					
	Meeting guidelines	42	21.43	0.89	0.8	0.62	0.4	0.61	0.4
	Takeaway Dinner								
	Once or less per month	132	20.45	reference					
	About once a week	117	24.79	1.28	0.4	1.35	0.4	1.31	0.5
	More than once a week	37	27.03	1.44	0.3	1.44	0.6	1.49	0.5
	Alcohol								
	Not meeting guidelines	159	24.53	reference					
	Meeting guidelines	127	21.26	0.83	0.6	0.71	0.3	0.64	0.2
	Tobacco								
	Smoker	32	21.88	reference					
	Non-smoker	254	23.23	1.08	0.8	0.71	0.5	0.61	0.4
	Physical Activity								
	Not meeting guidelines	178	20.79	reference					
	Meeting guidelines	108	26.85	1.40	**0.002**	1.42	0.1	1.48	0.2
	Sitting Time (hrs per day)								
	Weekday	286	-	0.94	0.1	0.99	0.9	1.00	0.9
	Weekend	286	-	0.80	**0.025**	0.78	0.053	0.75	**0.023**
	**Psychosocial Measures**								
5	Physical Functioning (SF12)	282	-	1.07	**<0.001**	1.08	**<0.001**	1.06	**0.004**
	Mental Functioning (SF12)	282	-	1.00	0.6	1.03	**0.022**	1.03	0.2
	**Physical Measures**^**d**^								
6	Blood Pressure (mmHg)								
	Systolic	273	-	0.97	**<0.001**	0.99	0.8	1.01	0.7
	Diastolic	273	-	0.95	**<0.001**	0.96	0.4	0.93	0.2
	Not meeting guidelines	56	7.14	reference					
	Meeting guidelines	217	26.73	4.74	**<0.001**				
	Heart rate (Mean, SD)	273	-	0.99	0.8	0.99	0.8	1.00	0.9
	Weight (Mean, SD)^e^	288	-	0.94	**<0.001**				
	Body Mass Index								
	Body Mass Index	288	-	0.64	**<0.001**	0.55	**<0.001**	0.51	**<0.001**
	Not meeting guidelines	238	15.13	reference					
	Meeting guidelines	50	60	8.42	**<0.001**				
	Waist circumference^f^								
	Waist circumference	288	-	0.91	**<0.001**				
	**Biomedical Measures (fasting)**^**d**^								
7	Total Cholesterol								
	Total Cholesterol	285	-	0.60	**0.001**	0.66	**0.018**	0.63	0.1
	Not meeting guidelines	89	14.61	reference					
	Meeting guidelines	196	26.53	2.11	**0.040**				
	Glucose								
	Glucose	285	-	1.40	0.5	2.60	1.0	3.00	1.0
	Not meeting guidelines	18	22.22	reference					
	Meeting guidelines	267	22.85	1.04	1.0				
	Triglycerides								
	Triglycerides	285	-	1.48	**0.001**	1.54	**0.012**	3.12	0.7
	Not meeting guidelines	73	16.44	reference					
	Meeting guidelines	212	25	1.69	0.1				
	**Predicted risk scores**^**d**^								
8	Cardiovascular disease risk (next 10 years)								
	CVD risk (continuous)	267	-	0.93	0.1	0.89	0.1	1.26	0.2
	Low-risk	227	23.35	reference					
	Intermediate-risk	33	12.12	0.45	0.2				
	High-risk	7	42.86	2.46	0.4				
	Diabetes risk (next 5 years)^f^								
	Diabetes risk (continuous)	285	-	0.88	**0.031**				
	Low-risk	67	40.3	reference					
	Intermediate-risk	173	19.08	0.35	**<0.001**				
	High-risk	45	11.11	0.19	**0.013**				
	**WORKPLACE CHARACTERISTICS**^**g**^								
9	Public ownership (vs. private)	288	21.77	0.89	0.6	1.30	0.4	1.31	0.6
	Outer city location (vs. inner city)	288	22.58	0.97	0.9	1.12	0.8	1.21	0.8
	**PROCESS MEASURES**								
	**Step data**^**d**^								
10	Step average per day	288	-	1.00	0.2	1.00	0.2	1.00	0.3
	<10,000 steps average (per day)	98	19.39	reference					
	Meeting ≥10,000 steps average (per day)	190	24.74	1.37	0.4				

^a ^Associated risk-factor subgroups adjusted only for baseline demographic confounders

^b ^Associated risk-factor subgroups adjusted for all potential predictors within the analysis Group and above

^c ^The reference group for this binary variable is 'no'. The reference group data is not shown.

^d ^Continuous variables chosen over categorical variables

^e ^Excluded due to the inclusion of body mass index (BMI)

^f ^Excluded from model as baseline WC was not considered as a predictor.

^g ^The reference group for this binary variable is shown in brackets. The reference group data is not shown.

Note: For participants not meeting WC guidelines at baseline

In participants who had a high-risk WC at baseline, participants who had completed tertiary education at baseline had a 2.4 cm larger reduction in WC when compared to participants who had not completed tertiary education at baseline. For every millimole per litre less of total cholesterol at baseline, a 0.9 cm greater loss in WC at four-months was observed.

### Predictors of improving waist circumference to meet low-risk guidelines at four-months

Between baseline and four-months, 22.9% of participants who had high-risk WC at baseline improved their WC enough to meet low-risk guidelines at four-months, Table 5. Through univariable analysis, new baseline variables associated with reducing WC to meet low-risk guidelines at four-months, included not participating in the program for appearance reasons, meeting fruit intake guidelines, meeting physical activity guidelines, less weekend sitting time, higher physical functioning, lower systolic and diastolic blood pressure, meeting guidelines for blood pressure, lower baseline weight, lower BMI, meeting BMI guidelines, smaller WC, lower total cholesterol, meeting total cholesterol guidelines, more elevated triglycerides and higher diabetes risk, Table 5.

**Table 5:** Comparison of baseline characteristics between high-risk and low risk waist circumference at baselinea

	Low-risk WC (Mean ± SD or Percentage)	High-risk WC (Mean ± SD or Percentage)	P-value
n	251	288	-
**WORKPLACE CHARACTERISTICS**^**b**^			
Public ownership (vs. private)	32.7	43.1	<0.001
Inner city location (vs. outer city)	70.9	67.7	0.4
**DEMOGRAPHICS**			
Age (year)	38 ± 10	43 ± 10	<0.001
Male	52.2	35.8	<0.001
Socio Economic Status (by SEIFA)			
Most Advantaged	37.5	33.1	0.3
Advantaged	40.6	42.5	
Disadvantaged	15.9	18.1	
Most Disadvantaged	6.0	6.3	
Completion of tertiary education	81.6	76.0	0.2
Occupation			
Professional	50.7	40.2	0.022
Associate professional	19.2	20.3	
Manager	17.9	21.1	
Clerical or Service	12.2	18.4	
Marital Status			
Married or de facto	67.5	69.9	0.6
Widowed, separated or divorced	8.1	9.1	
Never married	24.4	21.0	
**BASELINE MEASURES**			
Prior GCC^® ^Participation^c^	21.54	24.83	0.06
**Motivation for Participation**^**c**^			
Health	56.7	76.6	<0.001
To look my best	43.3	72.7	<0.001
Fitness	60.8	73.1	0.038
Colleagues	58.4	55.6	0.4
Friends or family	2.5	2.5	1.0
**Behavioural Measures**			
Self reported hypertension	10.3	24.6	0.001
Self reported diabetes	2.1	8.4	0.001
Fruit Intake (meeting guidelines)	28.5	34.3	0.5
Vegetable Intake (meeting guidelines)	14.2	14.7	0.9
Takeaway Dinner			
Once or less per month	46.3	46.2	0.9
About once a week	39.4	40.9	
More than once a week	14.2	12.9	
Alcohol (meeting guidelines)	39.8	44.4	0.1
Non tobacco smoker	92.7	88.8	0.2
Physical Activity (meeting guidelines)	41.2	37.8	0.018
Sitting Time (hrs per day)			
Weekday	8.3 ± 3.6	8.2 ± 3.6	0.8
Weekend	5.7 ± 3.1	4.0 ± 2.6	0.001
**Physical Measures**			
Systolic Blood Pressure (mmHg)	116.1 ± 14.0	120.9 ± 15.0	0.010
Diastolic Blood Pressure (mmHg)	77.2 ± 10.0	81.8 ± 10.1	0.001
Blood Pressure (meeting guidelines)	86.1	79.5	0.036
Heart rate (beats per minute)	67.3 ± 10.2	69.5 ± 9.3	0.007
Weight (kg)	68.7 ± 11.1	84.1 ± 15.4	<0.001
Body Mass Index (kg/m^2^)	23.6 ± 2.4	29.2 ± 4.5	<0.001
Body Mass Index (meeting guidelines)	69.1	17.4	<0.001
Waist circumference (cm)	79.4 ± 8.1	95.2 ± 10.2	<0.001
Waist circumference (meeting guidelines)	100.0	0.0	<0.001
**Biomedical Measures (fasting)**			
Total Cholesterol (mmol/L)	4.8 ± 0.9	5.0 ± 1.0	0.1
Total Cholesterol (meeting guidelines)	75.6	68.8	0.025
Glucose (mmol/L)	4.9 ± 0.7	5.2 ± 1.1	0.004
Glucose (meeting guidelines)	99.2	93.7	0.025
Triglycerides (mmol/L)	1.0 ± 0.5	1.3 ± 1.0	0.003
Triglycerides (meeting guidelines)	85.6	74.4	<0.001
**Predicted risk scores**			
Cardiovascular disease risk (next 10 years)			
CVD risk	2.9 ± 4.2	5.5 ± 5.2	0.001
High-risk	0.00	2.62	0.002
Intermediate-risk	5.06	12.36	
Diabetes risk (next 5 years)			
Diabetes risk	5.5 ± 3.3	9.3 ± 5.1	<0.001
High-risk	2.5	15.8	<0.001
Intermediate-risk	42.2	60.7	
**PROCESS MEASURES**			
**Step data**			
Steps average (per day)	11815 ± 3661	11491 ± 3690	0.5
Meeting 10,000 steps average (per day)	67.6	66.0	0.8
**FOUR-MONTH MEASURES**			
Waist circumference (cm)	79.3 ± 8.8	92.3 ± 11.32	<0.001
Waist circumference (meeting guidelines)	91.2	22.9	<0.001

^a^In participants who attended both baseline and four-month follow-up data collections.

^b ^The reference group for this binary variable is shown in brackets. The reference group data is not shown.

^c ^The reference group for this binary variable is 'no'. The reference group data is not shown

Although all participants in this sub-analysis had high-risk WC at baseline, a few were meeting guidelines for body composition when assessed by BMI. Within the high-risk WC group at baseline, participants who were meeting guidelines for BMI at baseline responded positively to the program and had 8.4 increased odds of improving their WC to meeting guidelines at four-months than participants not meeting baseline BMI guidelines, p < 0.001. Participants with low baseline diabetes type-2 risk had 5.4 increased odds of improving their WC to meeting guidelines at four-months than participants who were at high baseline diabetes risk, p = 0.013.

Within the high-risk baseline WC group, for every year increase in age at baseline, it was 3% less likely that the participant would improve their WC to meet low-risk guidelines at four-months. Employees participating in the program for reasons other than appearance had 2.6 increased odds of improving their WC to meet guidelines than employees participating for other reasons. Participants eating two or more pieces of fruit per day at baseline were 3.1 times more likely to improve their WC to meet guidelines than participants eating less than two pieces per day. For every hour decrease of weekend sitting time at baseline, it was 33% more likely that the participant would improve their WC to meet guidelines at four-months. For every BMI unit decrease at baseline, it was twice as likely that the participant would improve their WC to meet guidelines at four-months.

## Discussion

In this study analysing variables associated with waist circumference (WC) change following participation in a four-month, pedometer-based, workplace health program, employees with a high-risk WC at baseline experienced the greatest improvements in WC. Strong predictors of improved WC during the program for all employees and employees with high-risk baseline WC were having completed tertiary education, undertaking less baseline weekend sitting time and having lower total cholesterol at baseline. An additional predictor of improvement in WC for all employees was not consuming more than two standard alcoholic beverages in one occasion during the twelve months prior to baseline. Unique baseline predictors were identified for improving WC to meet guidelines at four-months and these included participating for reasons other than appearance, eating at least two serves of fruit per day, higher physical functioning and lower BMI.

Our finding that employees with a high-risk WC at baseline experienced the greatest improvements in WC confirms findings from a similar workplace health program evaluation [14]. However, the relationship between baseline WC and WC change seems to be contradictory to the relationships observed with the other predictors, which mainly indicated better health. The finding that employees with larger WC at baseline improved the most during the program may be because they had the greatest opportunity to improve. In addition, some unique variables associated with improved WC that indicated better health were restricted to the high-risk baseline WC group.

The finding that employees with better health benefited the most from the program may indicate that healthier employees may find it easier to make the small changes required for a visible outcome. Whilst others, who need to make a greater change, may need additional support or motivation that may not be available through a workplace health program alone. However, a horse-racing effect [26] may be also present and as we did not assess behavioural change status, employees who are defined as 'healthier' at baseline may actually have recently made positive changes and be on a pathway to reducing their health risks. Hence, the workplace health program could be supporting employees to continue making the healthier behavioural changes. An alternate theory is that the markers of better health may be indicators of better socio-economic status. However, when several socio-economic confounders were included in the model, only adequate fruit intake in the high-risk baseline WC sub-analysis became non-significant.

Regardless of the reasoning for the healthier employees responding better to the program, the result highlights a possible need for these programs to respond to the enrolees and encourage change based on their individual baseline characteristics. Program initiators may need to think innovatively about how to further promote change in employees with multiple baseline risk factors.

The strengths of this evaluation included the range and quality of measurements, the large sample size and the variety of sedentary occupations within the sample [15]. The main limitation is the lack of assessment and evaluation of program and workplace characteristics. We recommend that future health programs not only evaluate a wide range of participant predictors (behavioural, anthropological and biological), but also the program and workplace characteristics as predictors. Another limitation is that the potential selection bias associated with workplace recruitment, individual recruitment and participant retention [9,15]. Workplaces electing evaluation may have been more motivated as they conscripted to the GCC^®^2008 early, study participants may have been more motivated and/or have better baseline health and returning study participants had healthier baseline measures [9,15,27,28]. A healthier, more motivated cohort would be more likely to comply with the health program (overestimating the health benefits) but a greater proportion of a healthier cohort would already be meeting health guidelines at baseline (underestimating the general health benefits of participation due to ceiling effects) [9]. However, selection bias is unlikely to substantially affect the interrelationships between predictors and WC change. A potential limitation is not having an explicit measure of the potential regression to the mean effect for waist circumference. However, as waist circumference decreased over time in both the low and high baseline risk groups there was no evidence of the regression to the mean effect in this sample.

## Conclusions

While employees with high-risk WC at baseline experienced the greatest improvements in WC, the other predictors of WC improvement were generally indicators of better health at baseline. These results indicate that employees who started with better health, potentially due to lifestyle or recent behavioural changes, were more likely to respond positively to the program. The results from this paper can be used to inform employees during recruitment for workplace physical activity programs that there are benefits for participants who are in the healthier spectrum, as well as employees who have a higher WC risk. However, we suggest that these programs may need to promote additional support and motivation for participants who are at greatest health risk. We encourage future health program initiators to think innovatively about how to encourage all enrolees along the health spectrum to achieve clinically relevant improvement.

## Competing interests

This data was collected as part of the Global Corporate Challenge^® ^Evaluation Study conducted through Monash University. We would like to acknowledge Maximilian de Courten and Margaret Brand for their assistance during this process. We would like to acknowledge the Australian Research Council (ARC) and the Foundation for Chronic Disease Prevention™ in the Workplace, which is associated with the Global Corporate Challenge^®^, for partially funding this study. The study design; analysis and interpretation of data; the writing of the manuscript; and the decision to submit the manuscript for publication were solely at the discretion of the Monash researchers, independent of GCC^® ^or The Foundation's involvement. RFP, RW, HW, KB & AP are affiliated with Monash University. RFP is supported by an Australian Postgraduate Award and a Monash Departmental Scholarship. AP is funded by a VicHealth Public Health Fellowship. The authors declare that they have no further competing interests.

## Authors' contributions

RFP and AP undertook the study design and oversaw the data collection for the project. RFP takes responsibility for the integrity of the data and the accuracy of the data analysis. RFP, RW and AP contributed to the statistical data analysis. RFP, RW, HW, KB & AP contributed to the critical interpretation of the data. All authors contributed to the final version of the paper and have read, as well as, approved the final manuscript.

## Pre-publication history

The pre-publication history for this paper can be accessed here:

http://www.biomedcentral.com/1471-2458/11/824/prepub
